# Genetic Variants, Metabolic Dysfunction-Associated Fatty Liver Disease, and Major Health Outcomes in Older Adults

**DOI:** 10.3390/biomedicines13081977

**Published:** 2025-08-14

**Authors:** Daniel Clayton-Chubb, Ammar Majeed, William W. Kemp, Chenglong Yu, Peter W. Lange, Jessica A. Fitzpatrick, Robyn L. Woods, Andrew M. Tonkin, Andrew T. Chan, Mark R. Nelson, Joanne Ryan, Alexander D. Hodge, John S. Lubel, Hans G. Schneider, John J. McNeil, Stuart K. Roberts

**Affiliations:** 1 Department of Gastroenterology, Alfred Health, Melbourne 3004, Australia; a.majeed@alfred.org.au (A.M.); w.kemp@alfred.org.au (W.W.K.); jessica.fitzpatrick1@monash.edu (J.A.F.); johnlubel@icloud.com (J.S.L.); 2 Department of Medicine, School of Translational Medicine, Monash University, Melbourne 3004, Australia; 3 Department of Gastroenterology, Eastern Health, Melbourne 3128, Australia; alex.hodge@monash.edu; 4 Sheila Sherlock Liver Unit, Royal Free Hospital, London NW3 2QG, UK; 5 School of Public Health and Preventive Medicine, Monash University, Melbourne 3004, Australia; chenglong.yu@monash.edu (C.Y.); robyn.woods@monash.edu (R.L.W.); andrew.tonkin@monash.edu (A.M.T.); mark.nelson@utas.edu.au (M.R.N.); joanne.ryan@monash.edu (J.R.); h.schneider@alfred.org.au (H.G.S.); john.mcneil@monash.edu (J.J.M.); 6 Department of Geriatrics and General Medicine, Werribee Mercy Hospital, Werribee 3030, Australia; aprofpeterlange@outlook.com; 7 Department of Aged Care and Medicine, The Royal Melbourne Hospital, Melbourne Medical School, The University of Melbourne, Parkville 3050, Australia; 8 Clinical and Translational Epidemiology Unit, Massachusetts General Hospital, Boston, MA 02114, USA; achan@mgh.harvard.edu; 9 Division of Gastroenterology, Massachusetts General Hospital, Boston, MA 02114, USA; 10 Harvard Medical School, Harvard University, Boston, MA 02115, USA; 11 Menzies Institute for Medical Research, University of Tasmania, Hobart 7001, Australia; 12 School of Health and Biomedical Science, Royal Melbourne Institute of Technology, Melbourne 3000, Australia; 13 Department of Medicine, Eastern Clinical School, Monash University, Melbourne 3128, Australia; 14 Department of Pathology, Alfred Health, Melbourne 3004, Australia

**Keywords:** single-nucleotide polymorphisms, MAFLD, MASLD, fatty liver disease, older adults, epidemiology, genetics, cardiovascular disease, mortality, ageing

## Abstract

**Background and Aims**: Multiple genetic variants have been associated with disease prevalence and outcomes in middle-aged people with metabolic dysfunction-associated fatty liver disease (MAFLD). However, genetic studies in older adults have been lacking. We aimed to understand their clinical relevance in healthy older persons. **Methods**: A secondary analysis of the ASPREE (ASPirin in Reducing Events in the Elderly) randomized trial involving community-dwelling older adults ≥ 70 years without prior cardiovascular disease events or life-limiting illness at enrolment. The Fatty Liver Index (FLI) was used to identify MAFLD at baseline. We assessed the associations between six previously reported MAFLD-associated genetic variants with prevalent MAFLD at baseline, and the associations of these variants with cardiovascular disease events and all-cause mortality. **Results**: A total of 8756 participants with genetic data were stratified according to the FLI, with 3310 having MAFLD at baseline. The follow-up was for a median of 8.4 (IQR 7.3–9.5) years. Variants in two genes (*GCKR* and *HSD17B13*) were associated with prevalent MAFLD (*p* < 0.05); *PNPLA3*, *TM6SF2*, *LYPLAL1*, and *MBOAT7* were not. *PNPLA3*, *TM6SF2*, *HSD17B13*, *GCKR*, and *LYPLAL1* were *not* associated with major adverse cardiovascular events (MACEs) or mortality in the overall cohort or in participants with MAFLD during the follow-up (all *p* > 0.05). Within the MAFLD group, homozygosity for the rs641738 C > T variant in the *MBOAT7* gene was associated with a reduced risk of MACEs (HR 0.68 [95% CI 0.48–0.97]), but not all-cause mortality (HR 1.14 [95% CI 0.89–1.47]). This protective association remained significant after adjusting for multiple key covariates (aHR 0.64 [95% CI 0.44–0.92]). The results were similar when using the metabolic dysfunction-associated steatotic liver disease definition rather than MAFLD. **Conclusions**: The rs641738 C > T variant in *MBOAT7* may confer protection against MACEs in older adults with MAFLD, independent of other clinical risk factors. Further validation using external cohorts is needed.

## 1. Introduction

Metabolic dysfunction-associated fatty liver disease (MAFLD) is characterized by ≥5% of hepatocytes containing fat alongside one or more of diabetes mellitus (T2DM), overweight/obesity, and/or ≥2 cardiometabolic comorbidities [1]. Previous work from our group and others has linked MAFLD with atherosclerotic cardiovascular disease (CVD) [2,3,4], liver cancer [5], and, in some studies, all-cause mortality [3,6]. These findings are of interest due to the growing prevalence of MAFLD globally [7]. However, there are discrepant findings regarding the outcomes in older adults, with some suggestions that the risks of MAFLD are attenuated in those over 70 years of age [8,9], perhaps due to survival bias.

Given the significant prevalence of MAFLD in middle-aged and older adults (30–40%) [3,10], driven in part by the rising prevalence of overweight/obesity [11] and T2DM [12], there is an increasing focus on risk-stratifying adults with MAFLD, in terms of those at greater risk of adverse health outcomes, to assist with focusing medical interventions on those most likely to benefit [13]. One proposed component of personalized risk assessment includes the evaluation of genetic variants that may predispose individuals to MAFLD [13]. While MAFLD is a polygenic disease, a multitude of individual genetic variants have been associated with the risk of MAFLD or varied related outcomes in those living with MAFLD. These variants include patatin-like phospholipase domain-containing 3 (*PNPLA3*) rs738409 C > G, Transmembrane 6 Superfamily Member 2 (*TM6SF2*) rs58542926 C > T, 17β-hydroxysteroid dehydrogenase type 13 (*HSD17B13*) rs72613567 T > TA, Glucokinase Regulatory Protein (*GCKR*) rs1260326 C > T, Lysophospholipase-Like 1 (*LYPLAL1*) rs12137855 C > T, and Membrane-Bound O-Acyltransferase Domain-Containing 7 (*MBOAT7*) rs641738 C > T [13,14,15]. Interestingly, while *PNPLA3* and *TM6SF2* have been associated with an increased risk of hepatic steatosis and steatohepatitis, they have also been previously associated with a reduced risk of CVD in adults [13]. However, the implications of having one of these genetic variants on outcomes in later life is understudied. It is unknown whether healthy older persons are resistant to the phenotypic impacts of these variants (i.e., an altered risk of having MAFLD), as well as whether these variants impact major clinical outcomes in older persons with MAFLD.

As such, our present study sought to examine the associations between known MAFLD-associated genetic variants and both prevalent MAFLD and key outcomes in older people. The objective of our study was to examine whether there may be a role for genetic risk stratification in predicting MAFLD development and MAFLD-related outcomes later in life. Specifically, we aimed to evaluate the prevalence and impact of previously described fatty liver disease-related genetic variants on mortality and CVD in older adults with MAFLD. Additionally, while recent Australian [16,17] and Asia–Pacific [18] guidelines use the MAFLD definition, the more recent metabolic dysfunction-associated steatotic liver disease (MASLD) [19] is used more commonly in Europe and the USA. As such, we additionally performed the same analyses for the ASPREE participants with MASLD.

## 2. Methods

### 2.1. Study Population

**Figure 1:**
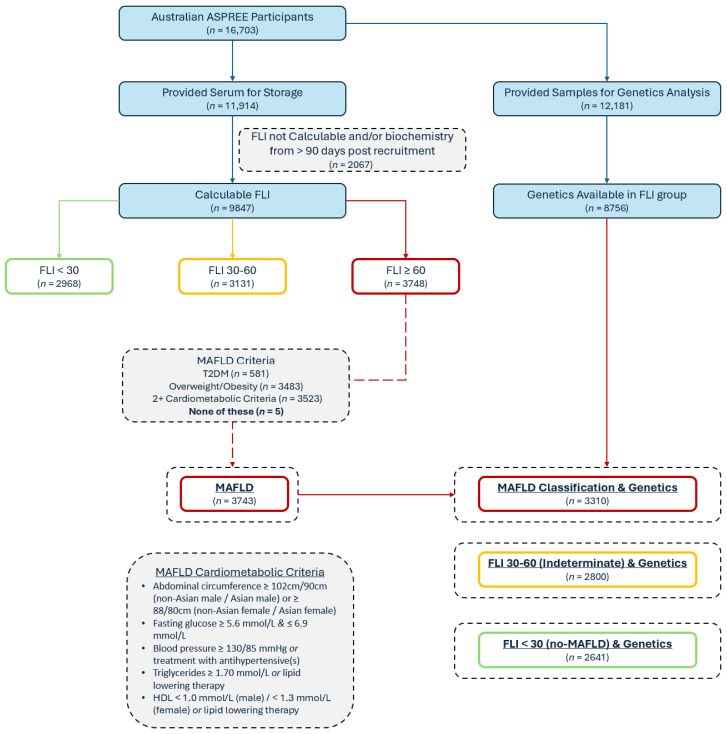
Baseline participant classification flow diagram; FLI = Fatty Liver Index; MAFLD = metabolic dysfunction-associated fatty liver disease.

The study design and findings from the main ASPirin in Reducing Events in the Elderly (ASPREE) trial have been previously reported in detail [20,21,22]. We performed an analysis of the Australian participants who provided serum [23] to allow for calculation of the Fatty Liver Index (FLI) [24]. The participants from the USA were unable to be analyzed as they did not provide serum for a baseline steatotic liver disease case identification. In summary, between 2010 and 2014, ASPREE recruited 16,703 Australian participants via primary care who were aged ≥ 70 years without dementia, prior cardiovascular disease events, or a life expectancy of less than five years. The participants were randomly assigned 1:1 to 100 mg of enteric-coated aspirin or matched placebo with an annual follow-up and medical records review (Figure 1).

The primary outcome of the ASPREE trial was disability-free survival. Major adverse cardiovascular events (MACEs), a composite of fatal coronary heart disease, nonfatal myocardial infarction, and fatal or nonfatal stroke, were among the adjudicated endpoints. The observational follow-up continued post-ASPREE with the ASPREE-eXTension (ASPREE-XT) cohort study [25]. This study used the data from up to the XT04 timepoint (approximately 4–5 years of ASPREE-XT follow-up).

The initial ASPREE trial was approved by the Monash University Human Research Ethics Committee (MUHREC) (IRB00002519; ethics #2006/745MC) and other allied institutions’ ethics committees. In Australia, the Alfred Hospital Ethics Committee (ethics #HREC/17/Alfred/198) oversees the ASPREE-XT project as the primary site approver. The ASPREE trial and ASPREE-XT cohort study are registered with ClinicalTrials.gov (NCT01038583) and the International Standard Randomised Controlled Trial Number Registry (ISRCTN83772183), and have been conducted in accordance with the Declaration of Helsinki.

### 2.2. Participant Assessment and Laboratory Data

At baseline and during the follow-up, in-person/phone call interviews and assessments by trained research staff, blinded to the randomized trial medication allocation, collected the following data: (a) self-reported information on medical history and lifestyle (including alcohol use); (b) anthropometry and markers of physical function (including BMI, abdominal circumference, blood pressure, and pulse rate); and (c) laboratory parameters via local pathology collection centers. The specific questionnaires included the Modified Mini-Mental State Examination (3MS) [26] (repeated every two years) and the Center for Epidemiologic Studies Depression Scale (CES-D-10) [27,28].

The initial baseline data included fasting triglycerides and other lipids, creatinine, and fasting glucose, which were repeated annually. In addition, as part of the ASPREE Healthy Ageing Biobank sub-study, 11,914 Australian participants provided blood samples for storage and later analysis. These samples were collected, stored, and subsequently used for biochemical analysis to determine the gamma-glutamyltransferase (GGT), amongst other measures. The analysis of the Healthy Ageing Biobank serum was performed centrally at Alfred Health Pathology using an Abbott Alinity ci analyzer and Abbott reagents (Abbott Diagnostics, Macquarie Park, NSW, Australia).

### 2.3. Identifying MAFLD

As previously described, the FLI [24] was calculated for the ASPREE cohort [9,29,30] using the BMI, abdominal circumference, triglycerides, and GGT in the participants for whom complete data were available and who had blood collected within 90 days of their baseline visit. Although the FLI was originally used and validated for identifying non-alcoholic fatty liver disease, it has also been subsequently validated and used in MAFLD [4,31] and in older adults [32]. The possible FLI scores range from 0 to 100; a score of ≥60 represents hepatic steatosis [24]. To identify a MAFLD subgroup, the participants with an FLI ≥ 60 were classified as having MAFLD if they had the following: DM, overweight/obesity, and/or two or more requisite cardiometabolic comorbidities irrespective of alcohol or medication consumption [1]. Individuals with an FLI of <30 were considered no-MAFLD comparators (Figure 1). To identify those with MASLD, an FLI ≥ 60 and the usual criteria were applied, as previously performed in the ASPREE cohort [9,30,33,34], including the exclusion of steatogenic medications and/or excess alcohol (14 or more drinks per week for women, 21 or more drinks per week for males). These exclusions are the most clinically important differences between the MASLD and MAFLD definitions.

### 2.4. Baseline Characteristics and Cardiometabolic Comorbidities

Obesity was defined as ≥30 kg/m^2^ [1]. An elevated abdominal circumference [35] was defined as ≥102 cm for males, and ≥88 cm for females. Hypertension was defined as a systolic blood pressure ≥ 140 mmHg, a diastolic blood pressure ≥ 90 mmHg, and/or the use of antihypertensive medication(s) [36]. DM was defined as one or more of self- or physician-reported DM, a fasting glucose ≥ 7.0 mmol/L, and/or the prescription of hypoglycemic medication(s) [37]. The HbA1c data was not available in ASPREE. Dyslipidemia was defined as one or more of the following: total cholesterol ≥ 6.2 mmol/L, LDL cholesterol ≥ 4.1 mmol/L, HDL cholesterol < 1.0 mmol/L, triglycerides > 2.3 mmol/L, and/or use of an HMG-CoA reductase inhibitor or a fibrate. The cardiometabolic abnormalities required for a diagnosis of MAFLD have been previously published, and included a BMI ≥ 25 kg/m^2^ (or BMI ≥ 23 kg/m^2^ in Asians); self-described DM, or a fasting blood glucose ≥ 7.0 mmol/L, or drug treatment for DM; or two or more of elevated abdominal circumference, fasting blood glucose ≥ 5.6 mmol/L, blood pressure ≥ 130/85 mmHg or treatment with an antihypertensive agent, triglycerides ≥ 1.70 mmol/L or lipid-lowering therapy, and low HDL cholesterol (≤1.0 mmol/L in males, ≤1.3 mmol/L in females) or lipid-lowering therapy [19]. Similarly, the criteria for MASLD were used as previously published, including a BMI ≥ 25 kg/m^2^ (or BMI ≥ 23 kg/m^2^ in Asians); elevated abdominal circumference (with sex- and ethnicity-specific cut-offs); self-described DM, or a fasting blood glucose ≥ 5.6 mmol/L, or drug treatment for DM; blood pressure ≥ 130/85 mmHg or treatment with an antihypertensive agent; and triglycerides ≥ 1.70 mmol/L, or low HDL cholesterol (≤1.0 mmol/L in males, ≤1.3 mmol/L in females), or lipid-lowering therapy [19]. As noted above, the HbA1c data is not available in ASPREE.

### 2.5. Genetics

The DNA samples provided by the ASPREE participants were genotyped using an Axiom 2.0 Precision Medicine Diversity Array (Thermo Fisher Scientific, Fresno, CA, USA), covering >850,000 markers selected for high genomic coverage based on the 1000 Genomes Project Phase III. Variant calling was performed using a custom pipeline aligned to the GRCh38 reference genome. Genotype imputation was conducted using the TOPMed Imputation Server with the TOPMed-r2 reference panel [38,39]. Variants with a low imputation quality (R^2^ < 0.3) were excluded, in line with our previous studies [40,41]. In this study, we focused on six variants previously associated with MAFLD [42,43,44]: *PNPLA3* rs738409 C > G, *TM6SF2* rs58542926 C > T, *HSD17B13* rs72613567 T > TA, *GCKR rs1260326* C > T, *LYPLAL1* rs12137855 C > T, and *MBOAT7* rs641738 C > T. Heterozygote and homozygote carriers were considered separately for the primary analysis, and grouped for the supplementary analyses. All the participants in this study were of European ancestry based on their genotype data.

### 2.6. Defining Outcomes

The study adjudication process for validating MACEs, death, and cause of death in the overall ASPREE study was previously described [45]. Briefly, each case was independently reviewed by two clinical experts with a third adjudicator resolving discordance. Per the original ASPREE publication [22] based on the World Health Organization criteria, MACE was defined as a composite of fatal coronary heart disease (excluding death from heart failure), nonfatal acute myocardial infarction (AMI), or fatal/nonfatal ischemic stroke [22]. Similarly, the ascertainment and adjudication of the death data in ASPREE was previously described [21]. A death was usually identified either during a routine trial activity or through a relative/next-of-kin notifying the researchers. All deaths required confirmation from two independent sources. The staff also performed Ryerson Index linkages, a community-maintained volunteer-compiled register based on death notices and obituaries. Once a death was confirmed, the clinical details were sought from the relevant facilities and practitioners. Adjudication occurred via collated case presentations to at least two adjudicators (one USA based and one Australia based); discordance was resolved via consensus. The follow-up and adjudication of these endpoints has continued following the cessation of the randomized interventional trial.

### 2.7. Statistical Analysis

The baseline data were compared using a one-way ANOVA or two-tailed Student’s t-test (for continuous variables) or a chi-squared test (for categorical variables) for the MAFLD vs. no-MAFLD group, and carrier status for the various genetic variants was evaluated within the MAFLD sub-group. The co-primary outcomes were whether previously described genetic variants increased the hazard of MACE or all-cause mortality in older adults with MAFLD. An exploratory analysis evaluated the associations between these genetic variants with MACE and all-cause mortality in those without definite MAFLD (FLI < 60). Additionally, the analyses were repeated using the MASLD definition.

In the initial analysis, Cox proportional hazards regression models were used to evaluate the outcomes, adjusting first for the top 20 genetic principal components to account for population structure, followed by further adjustments for age and sex. Finally, the model evaluating the risk of MACE was subsequently also adjusted for the variables known to be associated with atherosclerotic cardiovascular disease in the overall ASPREE cohort [46] as well as randomization to aspirin/placebo. These variables included age, sex, serum creatinine, HDL and non-HDL cholesterol, systolic blood pressure, the prescription of an antihypertensive(s), DM, and being a current smoker. The final model evaluating the risk of all-cause mortality was adjusted for the variables known to be associated with reduced disability-free survival in the ASPREE cohort [47], including age, sex, markers of depression (CES-D-10 ≥ 8) and cognitive function (3MS), gait speed, grip strength, DM, current smoking status, and eGFR. No models included BMI or triglycerides due to their inclusion in the FLI. A two-tailed *p*-value of < 0.05 was considered statistically significant. Statistical analyses were performed using Stata software v17.0 (StataCorp LLC, College Station, TX, USA). Neither the participants nor the public were involved in the study design or reporting of this research.

## 3. Results

### 3.1. Study Population and Genetic Variant Prevalence

Of the 16,703 Australian participants enrolled in ASPREE, 11,914 provided blood for the biobank, and 9847 participants had a calculable FLI using the laboratory results from the blood samples taken within 90 days post-recruitment (Figure 1). Of the group with a calculable FLI, 3748 (38.1%) had an FLI ≥ 60, and of these, 3743 met the criteria for MAFLD. For the genetic analysis, 12,181 of the 16,703 participants had genetic data available (72.9%), including 8751 of those with a calculable FLI, leaving 3310 (88.4%) of the MAFLD patients with known allele carrier status (Figure 1). Of the included 8751 participants, the mean age was 75.0 ± 4.2 years and 53.5% were female. Their mean BMI (± SD) was 28.0 ± 4.5 kg/m^2^. In total, 37.8% had a diagnosis of MAFLD. The general characteristics of the participants stratified by their FLI are shown in Table 1 (comparing no-MAFLD [FLI < 30], an indeterminate FLI [30–60], and MAFLD [FLI ≥ 60]). The follow-up for these participants comprised the trial duration (median of 4.7 years) and the ASPREE-XT continuation, for a total follow-up of a median of 8.4 (IQR 7.3–9.5) years.

There was no significant difference in the carrier frequency of the genetic variants investigated between the FLI categories for wildtype *PNPLA3*, wildtype *MBOAT7*, wildtype *TM6SF2*, and wildtype *LYPLAL1* (all *p* > 0.05) (Table 1). There were statistically significant differences in the *HSD17B13* rs72613567 T > TA homozygotes (6.8% MAFLD, 7.9% FLI 30–60, and 7.4% no-MAFLD; *p* = 0.010) and the *GCKR rs1260326* C > T homozygotes (17.6% MAFLD, 15.4% FLI 30–60, and 14.6% no-MAFLD; *p* = 0.005) (Table 1). There were no significant differences in the variant frequencies between the sexes (Table 2).

### 3.2. Major Adverse Cardiovascular Events

There were 283 cases of MACE in the MAFLD group (8.5%) over a median (IQR) follow-up period of 8.3 (7.3–9.4) years, corresponding to an event rate of 10.8 per 1000 person-years (Table 3).

None of the individual MAFLD-associated genetic variants was associated with an increased risk of MACE based on a univariate or adjusted analysis (Table 4). *PNPLA3* (rs738409 C > G), *TM6SF2* (rs58542926 C > T), *HSD17B13* (rs72613567 T > TA), *GCKR (rs1260326* C > T), and *LYPLAL1* (rs12137855 C > T) were not protective of MACE (Table 4).

A total of 44 of the 641 (6.9%) persons with *MBOAT7* rs641738 C > T homozygosity reached the MACE endpoint compared with 104 of the 929 (11.2%) rs641738 wildtype participants. *MBOAT7* rs641738 C > T homozygosity was associated with a reduced risk of MACE in MAFLD (HR 0.78 [95% CI 0.61–0.99]). These findings held true after adjusting for multiple known cardiovascular disease risk factors in this population [46] (aHR 0.64 [95% CI 0.44–0.92]) (Table 4). Additionally, *MBOAT7* rs641738 C > T heterozygosity was non-significantly associated with a reduced risk of MACE (aHR 0.81 [95% CI 0.62–1.05]).

The above results were unchanged by an exploratory analysis of the homozygous and heterozygous individuals grouped together. There were no significant associations between the grouped homozygous or heterozygous individuals in any of *PNPLA3*, *TM6SF2*, *HSD17B13*, *GCKR*, or *LYPLAL1* (all *p* > 0.05) (Table 5). At least one *MBOAT7* rs641738 C > T variant was associated with a reduced risk of MACE (aHR 0.76 [95% CI 0.59–0.97]) (Table 5).

### 3.3. All-Cause Mortality

In the subgroup of 3310 participants with MAFLD, there were 497 deaths (18.2 per 1000 person-years) (Table 3). From both the unadjusted and adjusted analysis, none of the individual variants in *PNPLA3*, *MBOAT7*, *TM6SF2*, *HSD17B13*, *GCKR*, or *LYPLAL1* were associated with the all-cause mortality (Table 6).

This held true in our exploratory analysis when evaluating heterozygous and homozygous status together (Table 7).

### 3.4. Genetic Variant Associations in Older Individuals Without MAFLD

Of the 5441 participants without MAFLD (FLI < 60), 380 (7.0%) reached a MACE endpoint during the median follow-up of 8.4 years (IQR 7.3–9.5 years). None of the previously identified genetic variants were either positively or negatively associated with MACE on the adjusted or unadjusted analysis of the non-MAFLD group, including *MBOAT7* (rs641738 C > T) homozygosity (aHR 0.92 [95% CI 0.68–1.25]) (Table 8). Similarly, of the 775 deaths over the median follow-up of 8.6 years (7.4–9.6 years), none of the six examined genetic variants had any significant association with mortality (Table 8).

### 3.5. Outcomes in the MASLD Group

There were 2624 individuals classified as MASLD (FLI ≥ 60 and the requisite cardiometabolic criteria) with available genetic data. Within this group, there were no meaningful differences in outcomes compared with the larger MAFLD group (Table 9). While *TM6SF2* (rs58542926 C > T) heterozygosity was associated with death in the MASLD group (HR 1.42 [95% CI 1.09–1.84]), this was not true when fully adjusted (aHR 1.31 [95% CI 0.99–1.71]) or in the homozygous group (aHR 0.91 [95% CI 0.22–3.71]). No other genetic variants were associated with death in the MASLD group (Table 9). 

Similar to the MAFLD subgroup, *MBOAT7* (rs641738 C > T) homozygosity was again associated with a reduced risk of MACE on both the unadjusted (HR 0.66 [95% CI 0.44–0.99]) and fully adjusted (aHR 0.59 [95% CI 0.38–0.90]) analyses. No other genetic variants were associated with either an increased or decreased risk of MACE.

## 4. Discussion

The impact of MAFLD on morbidity, mortality, and MACE in older adults is unclear, with divergent results across multiple studies [8,48,49,50]. Given these discrepancies in the literature, and that many guidelines discuss or recommend screening for MAFLD in at-risk adults [51,52,53], this study aimed to identify whether there were any significant relationships between six well-recognized MAFLD-related genetic variants with all-cause mortality or MACE in older adults. The main findings of our study are that, in older adults, *MBOAT7* (rs641738 C > T) homozygosity is associated with a reduced risk of MACE in older adults with MAFLD (but not in a population of older adults without MAFLD), but is not associated with mortality. Additionally, there are no apparent associations of the variants *PNPLA3* (rs738409 C > G), *TM6SF2* (rs58542926 C > T), *HSD17B13* (rs72613567 T > TA), *GCKR (rs1260326* C > T), or *LYPLAL1* (rs12137855 C > T) with death or MACE in older adults with or without MAFLD. These results are similar to those when using the MASLD definition. Our findings have important implications for considering—or not considering—the use of genetic risk stratification for MAFLD or MASLD in older adults.

Multiple studies have evaluated the impact of various single-nucleotide polymorphisms and genetic variants on MAFLD prevalence and outcomes in middle-aged adults [13,14,15]. These studies have shown different relationships between genetic variants and MAFLD, including associations with not only the likelihood but also the severity of disease (including histological markers of severity, such as fibrosis and steatohepatitis) [54]. However, in our study, none of the four gene variants previously shown to be associated with prevalent MAFLD in middle age (candidate variants in the *PNPLA3*, *TM6SF2*, *LYPLAL1*, and *MBOAT7* genes) was associated with a meaningfully increased risk of prevalent MAFLD. While previous data has shown that HSD17B13 (rs72613567 T > TA) is associated with a reduced risk of MAFLD, this was of only marginal significance in our cohort (Table 1). The sixth variant evaluated (*GCKR rs1260326* C > T) was associated with a marginally increased risk of prevalent MAFLD (Table 1).

Additionally, none of the six evaluated gene variants showed an increased risk of all-cause mortality in older adults with MAFLD, nor were they associated with an altered risk of mortality in the no-MAFLD population. This could possibly be due to the age and general health of the ASPREE participants, who were all over 70 years and free from known life-limiting illnesses, pre-existing atherosclerotic cardiovascular disease, and dementia. Additionally, cancer was the predominant cause of death in this population, perhaps limiting the predictive utility of these gene variants. Regardless, it is possible that the deleterious effects of these genotypes are seen in middle age, and our cohort exhibited a survival bias or resilience to adverse genotypes. Furthermore, the mechanisms of these genetic variants in MAFLD development and progression tend to be related to lipid droplet metabolism, very-low-density lipoprotein metabolism, and de novo lipogenesis [54]. It is possible that participants engaged enough in their health to enroll in a primary prevention study were more actively involved in health prevention in their middle years leading to a healthy survivorship bias, which may partly attenuate the risk conferred by these alleles. Interestingly, the putative protective effect of *HSD17B13* (rs72613567 T > TA) [54] on steatohepatitis and progressive liver disease was also not evident in the altered mortality rates in either the MAFLD or no-MAFLD group. This may be because of the attenuated risk between MAFLD and mortality in older adults [9,49], such that a reduced risk of MAFLD development has no mechanism by which to have an impact on rates of liver- or non-liver-related death.

Despite the previously identified associations between MAFLD and steatohepatitis with atherosclerotic cardiovascular disease, none of the examined variants in *PNPLA3* (rs738409 C > G), *TM6SF2* (rs58542926 C > T), *GCKR (rs1260326* C > T), *LYPLAL1* (rs12137855 C > T), or *HSD17B13* (rs72613567 T > TA) was associated with either an increased or decreased risk of MACE. This was the case in both the MAFLD and no-MAFLD groups, as well as the MASLD group. These findings are largely in keeping with other research in younger populations, where *PNPLA3* (rs738409 C > G) (despite having been previously associated with increased liver-related disease) is protective against cardiovascular disease. Interestingly, *MBOAT7* (rs641738 C > T) was inversely associated with MACE—even when adjusting for a multitude of risk factors—in both the MAFLD and MASLD cohorts, but not in the no-MAFLD cohort. Previous work has suggested that *MBOAT7* rs641738 C > T has a relatively minor impact on cardiovascular disease [55], in spite of its known mechanistic association with an increased accumulation of free arachidonic acid (a precursor of proinflammatory lipid mediators) [55]. However, in a smaller study of older Chinese persons, a trend towards *MBOAT7* (rs641738 C > T) homozygosity being protective for atherosclerotic CVD in NAFLD was also seen, though in that study this effect was also seen in the no-NAFLD group [56]. The mechanism of protection seen in our study is uncertain and merits further exploration, as it may yield novel therapeutic targets to reduce the risk of MACE in older adults with MAFLD. However, this may also be a chance finding and requires replication in larger cohorts.

### Strengths and Weaknesses

Our study has several strengths, including its size, the rigorous protocol-driven prospective data capture at enrolment and during follow-up, and the robust ascertainment and adjudication of the key endpoints. However, some limitations should be discussed. The FLI was used to determine MAFLD, rather than a liver biopsy or imaging. While the FLI has been previously validated as a population-based marker of hepatic steatosis when compared to abdominal ultrasound-diagnosed fatty liver disease in a similar population to ours [32], an abdominal ultrasound itself is not the most sensitive modality for diagnosing hepatic steatosis. While there is a lack of data directly correlating the FLI to biopsy-proven MAFLD, these results broadly support our categorization of MAFLD vs. no-MAFLD. The FLI is also endorsed by the Gastroenterological Society of Australia MAFLD guidelines for use in epidemiological studies [17]. Additionally, the FLI may misclassify lean MAFLD, reducing the ability to extrapolate our findings to that group (or requiring alternative FLI cut-offs [57,58]). When considering the requisite cardiometabolic criteria for classifying individuals as MAFLD (or MASLD), ASPREE does not include HbA1c data, potentially impacting the ability to accurately apply these classification schemas. However, very few of the FLI ≥ 60 (steatotic) ASPREE participants did not meet the MAFLD criteria (5/3748, 0.13%), implying that the vast majority met the other cardiometabolic criteria. Additionally, the ASPREE population was a relatively healthy community-dwelling cohort and care should therefore be taken in extrapolating this data to wider older adult populations. However, because of this, our study population has a presumed longer life expectancy, such that risk stratifying those with MAFLD could be considered worthwhile despite their biological age, and that understanding the utility of personalized genetic testing is important for determining age-appropriate recommendations in clinically relevant populations. We did not adjust for multiple comparisons, though this is in keeping with the modern literature [59] on the topic, and our results became marginally more significant with adjustments for multiple confounders, perhaps increasing the possibility of an independent association. Importantly, our study population was not ethnically diverse, thereby limiting the capacity to generalize the findings to other non-European ethnicities in terms of both the overall relationship (or lack thereof), as well as the effect sizes. The confirmation of these findings using other large datasets would be valuable. Finally, as previously mentioned, MAFLD is a polygenic disease—the development of a polygenic risk score may improve risk stratification in the future.

## 5. Conclusions

In this sub-study of a very large randomized–controlled trial involving relatively healthy community-dwelling adults aged ≥ 70 years, there were only minor associations between two candidate variants (*GCKR* rs1260326 C > T and HSD17B13 rs72613567 T > TA) and prevalent MAFLD, and no association with four others (*PNPLA3*, *TM6SF2*, *LYPLAL1*, and *MBOAT7*). Additionally, there was no association between these candidate variants in the *PNPLA3*, *TM6SF2*, *HSD17B13*, *GCKR*, *LYPLAL1,* and *MBOAT7* genes with all-cause mortality in those with or without MAFLD. There was an association between *MBOAT7* (rs641738 C > T) homozygosity and a reduced risk of major adverse cardiovascular events—even when adjusting for age, sex, and multiple cardiovascular risk factors—in those with MAFLD or MASLD, but not in those without MAFLD. While these findings require replication in other cohorts, they may help inform the consideration of personalized risk stratification using genomics for older adults with MAFLD.

## Figures and Tables

**Table 1:** Baseline characteristics of Australian ASPREE participants classified by FLI and with genetics data.

Characteristic	All Participants	Non-MAFLD (FLI < 30)	FLI 30–60	MAFLD (FLI ≥ 60)	*p*-Value
Number of Participants	8751	2641 (30.2%)	2800 (32.0%)	3310 (37.8%)	
Sex (male)	4066 (46.5%)	820 (31.0%)	1437 (51.3%)	1809 (54.7%)	<0.001 ^a^
Age (years) (mean ± SD)	75.0 ± 4.2	75.3 ± 4.4	75.2 ± 4.4	74.5 ± 3.8	<0.001 ^b^
Alcohol Use					0.778 ^a^
Current Drinker (*n*, %)	6977 (79.7%)	2102 (79.6%)	2236 (79.9%)	2639 (79.7%)	
Former Drinker (*n*, %)	404 (4.6%)	117 (4.4%)	123 (4.4%)	164 (5.0%)	
Never Drinker (*n*, %)	1370 (15.7%)	422 (16.0%)	441 (15.8%)	507 (15.3%)	
Weight (kg) (mean ± SD)	76.12 ± 14.03	64.07 ± 8.69	75.75 ± 8.68	88.44 ± 12.16	<0.001 ^b^
BMI (kg/m^2^) (mean ± SD)	28.0 ± 4.5	23.9 ± 2.4	27.3 ± 2.2	31.8 ± 4.0	<0.001 ^b^ <0.001 ^a^
BMI Category [1] *					
Underweight	39 (0.4%)	39 (1.3%)	0 (0.0%)	0 (0.0%)	
Healthy Weight	2187 (25.0%)	1765 (66.8%)	388 (13.9%)	34 (1.0%)	
Overweight	4052 (46.3%)	821 (31.1%)	2088 (74.6%)	1143 (34.5%)	
Obese	2473 (28.3%)	16 (0.6%)	324 (11.6%)	2133 (64.4%)	
Waist Circumference (cm) (mean ± SD)	97.0 ± 12.5	84.3 ± 7.8	96.3 ± 6.4	107.8 ± 9.3	<0.001 ^b^
Elevated Abdominal Circumference [35] (*n*, %) ^†^	4989 (57.0%)	428 (16.2%)	1569 (56.0%)	2992 (90.4%)	<0.001 ^a^
Hypertension (*n*, %) ^¶^	5243 (59.9%)	1332 (50.4%)	1679 (60.0%)	2232 (67.4%)	<0.001 ^a^
Diabetes Mellitus (*n*, %) ^§§^	817 (9.3%)	95 (3.6%)	199 (7.1%)	523 (15.8%)	<0.001 ^a^
Dyslipidemia (*n*, %) **	4782 (54.6%)	1123 (42.5%)	1512 (54.0%)	2147 (64.9%)	<0.001 ^a^
Use of HMG-CoA Reductase Medication	2984 (34.1%)	677 (25.6%)	945 (33.8%)	1362 (41.1%)	<0.001 ^a^
Use of Fibrate Medication	76 (0.9%)	10 (0.4%)	18 (0.6%)	48 (1.5%)	<0.001 ^a^
Smoking History (*n*, %)					<0.001 ^a^
Never Smoked	4865 (55.6%)	1656 (62.7%)	1557 (55.6%)	1652 (49.9%)	
Former Smoker	3610 (41.3%)	896 (33.9%)	1150 (41.1%)	1564 (47.3%)	
Current Smoker	276 (3.2%)	89 (3.4%)	93 (3.3%)	94 (2.8%)	
Laboratory Values ^¶¶^					
GGT (U/L) (median [IQR])	21 (16–32)	16 (13–21)	21 (16–28)	29 (21–45)	<0.001 ^c^
ALT (U/L) (median [IQR])	18 (14–23)	16 (13–19)	18 (14–22)	21 (16–28)	<0.001 ^c^
AST (U/L) (median [IQR])	21 (18–24)	20 (18–24)	20 (18–24)	21 (18–26)	<0.001 ^c^
Total Cholesterol (mmol/L) (mean ± SD)	5.3 ± 1.0	5.4 ± 0.9	5.3 ± 1.0	5.2 ± 1.0	0.042 ^b^
Non-HDL-C (mmol/L) (mean ± SD)	3.7 ± 0.9	3.5 ± 0.9	3.7 ± 0.9	3.8 ± 1.0	<0.001 ^b^
HDL-C (mmol/L) (mean ± SD)	1.6 ± 0.5	1.8 ± 0.5	1.6 ± 0.4	1.4 ± 0.4	<0.001 ^b^
Triglycerides (mmol/L) (mean ± SD)	1.3 ± 0.7	0.9 ± 0.3	1.2 ± 0.5	1.7 ± 0.8	<0.001 ^b^
Renal Function					
eGFR (mL/min/1.73 m^2^) (mean ± SD)	73.0 ± 13.4	74.8 ± 12.7	72.6 ± 13.1	71.7 ± 14.1	<0.001 ^b^
Creatinine (mcmol/L) (mean ± SD)	80.0 ± 0.22	74.6 ± 16.2	81.1 ± 18.7	83.4 ± 20.7	<0.001 ^b^
Chronic Kidney Disease ^§§§^ (*n*, %)	1533 (17.5%)	364 (13.8%)	478 (17.1%)	691 (20.9%)	<0.001 ^a^
MAFLD variants					
PNPLA3 (rs738409 C > G) (*n*, %)					0.613 ^a^
Wildtype	5292 (60.5%)	1593 (60.3%)	1726 (61.6%)	1973 (59.6%)	
Heterozygous	3047 (34.8%)	924 (35.0%)	946 (33.8%)	1177 (35.6%)	
Homozygous	412 (4.7%)	124 (4.7%)	128 (4.6%)	160 (4.8%)	
MBOAT7 (rs641738 C > T) (*n*, %)					0.569 ^a^
Wildtype	2779 (31.8%)	862 (32.6%)	884 (31.6%)	1033 (31.2%)	
Heterozygous	3405 (49.2%)	1269 (48.0%)	1400 (50.0%)	1636 (49.4%)	
Homozygous	1667 (19.0%)	510 (19.3%)	516 (18.4%)	641 (19.4%)	
TM6SF2 (rs58542926 C > T) (*n*, %)					0.762 ^a^
Wildtype	7446 (85.1%)	2257 (85.5%)	2393 (85.5%)	2796 (84.5%)	
Heterozygous	1255 (14.3%)	369 (14.0%)	393 (14.0%)	493 (14.9%)	
Homozygous	50 (0.6%)	15 (0.6%)	14 (0.5%)	21 (0.6%)	
HSD17B13 (rs72613567 T > TA) (*n*, %)					0.010 ^a^
Wildtype	4558 (52.1%)	1366 (51.7%)	1514 (54.0%)	1680 (50.8%)	
Heterozygous	3545 (40.5%)	1074 (40.7%)	1066 (38.1%)	1405 (42.4%)	
Homozygous	648 (7.4%)	201 (7.6%)	222 (7.9%)	225 (6.8%)	
GCKR (rs1260326 C > T) (*n*, %)					0.005 ^a^
Wildtype	3095 (35.4%)	988 (37.4%)	983 (35.1%)	1124 (34.0%)	
Heterozygous	4257 (48.6%)	1267 (48.0%)	1386 (49.5%)	1604 (48.5%)	
Homozygous	1399 (16.0%)	386 (14.6%)	431 (15.4%)	582 (17.6%)	
LYPLAL1 (rs12137855 C > T) (*n*, %)					0.858 ^a^
Wildtype	5394 (61.6%)	1623 (61.5%)	1714 (61.2%)	2057 (62.1%)	
Heterozygous	2994 (33.6%)	897 (34.0%)	945 (33.8%)	1102 (33.3%)	
Homozygous	413 (4.7%)	121 (4.6%)	141 (5.0%)	151 (4.6%)	

* Underweight = BMI < 18.5 kg/m^2^; healthy weight = BMI 18.5–22.9 kg/m^2^ (Asian) or 18.5–24.9 kg/m^2^ (non-Asian); overweight = BMI 23–24.9 kg/m^2^ (Asian) or 25–29.9 kg/m^2^ (non-Asian); obese = BMI ≥ 25 kg/m^2^ (Asian) or ≥30 kg/m^2^ (non-Asian). ^†^ Elevated abdominal circumference = if Asian, ≥88 cm (males) and ≥80 cm (females); if non-Asian, ≥102 cm (males) and ≥90 cm (females). ^¶^ Defined as systolic blood pressure ≥140 mmHg and/or diastolic blood pressure ≥90 mmHg and/or prescription of at least one antihypertensive medication at baseline. ^§§^ Defined as one or more of the following: (a) self-reported diabetes mellitus, (b) prescription of at least one glucose-lowering therapy at baseline, (c) fasting blood glucose of ≥7.0 mmol/L. ** Defined as one or more of the following: total cholesterol ≥ 6.2 mmol/L, LDL cholesterol ≥ 4.1 mmol/L, HDL cholesterol < 1.0 mmol/L, triglycerides > 2.3 mmol/L, taking an HMG-CoA reductase inhibitor or a fibrate. ^¶¶^ GGT is gamma-glutamyltransferase, ALT is alanine aminotransferase, AST is aspartate aminotransferase. ^§§§^ Defined as eGFR < 60 mL/min/1.73 m^2^ and/or a urine albumin–creatinine ratio of >25 mg/g (males) or >35 mg/g (females). ^a^ Chi-square *p*-value; ^b^ one-way ANOVA *p*-value; ^c^ Kruskal–Wallis *p*-value.

**Table 2:** Associations between sex and MAFLD-related genetic variants (homozygotes and heterozygotes grouped).

MAFLD Genetic Variants	Female (*n* = 4685)	Male (*n* = 4066)	*p*-Value
PNPLA3 (rs738409 C > G)	1869 (39.9%)	1590 (39.1%)	0.452 ^a^
MBOAT7 (rs641738 C > T)	3221 (68.8%)	2751 (67.7%)	0.273 ^a^
TM6SF2 (rs58542926 C > T)	708 (15.1%)	597 (14.7%)	0.574 ^a^
HSD17B13 (rs72613567 T > TA)	2284 (48.8%)	1909 (47.0%)	0.093 ^a^
GCKR (rs1260326 C > T)	3028 (68.5%)	2628 (64.6%)	0.249 ^a^
LYPLAL1 (rs12137855 C > T)	1808 (38.6%)	1549 (38.1%)	0.635 ^a^

^a^ Chi-square *p*-value.

**Table 3:** Relationship between baseline characteristics and MACE and all-cause mortality in Australian ASPREE participants with MAFLD.

Outcome in the MAFLD Group	No MACE	MACE	*p*-Value	No Death	Death	*p*-Value
Number of participants	3027	283		2813	497	
Sex (male)	1619 (53.5%)	190 (67.1%)	**<0.001 ^a^**	1505 (53.5%)	304 (61.2%)	**0.002 ^a^**
Age (years) (mean ± SD)	74.4 ± 3.8	75.9 ± 4.5	**<0.001 ^b^**	74.2 ± 3.5	76.5 ± 4.8	**<0.001 ^b^**
MAFLD variants						
PNPLA3 (rs738409 C > G) (*n*, %)			0.494 ^a^			0.570 ^a^
Wildtype	1795 (59.3%)	178 (62.9%)		1669 (59.3%)	304 (61.2%)	
Heterozygous	1085 (35.8%)	92 (32.5%)		1004 (35.7%)	173 (34.8%)	
Homozygous	147 (4.9%)	13 (4.6%)		140 (5.0%)	20 (4.0%)	
MBOAT7 (rs641738 C > T) (*n*, %)			0.062 ^a^			0.620 ^a^
Wildtype	929 (30.7%)	104 (36.7%)		879 (31.2%)	154 (31.0%)	
Heterozygous	1501 (49.6%)	135 (47.7%)		1395 (49.6%)	239 (48.1%)	
Homozygous	597 (19.7%)	44 (15.5%)		537 (19.1%)	104 (20.9%)	
TM6SF2 (rs58542926 C > T) (*n*, %)			0.583 ^a^			0.207 ^a^
Wildtype	2563 (84.7%)	233 (82.3%)		2389 (84.9%)	407 (81.9%)	
Heterozygous	445 (14.7%)	48 (17.0%)		406 (14.4%)	87 (17.5%)	
Homozygous	19 (0.6%)	2 (0.7%)		18 (0.6%)	3 (0.6%)	
HSD17B13 (rs72613567 T > TA) (*n*, %)			0.645 ^a^			0.618 ^a^
Wildtype	1529 (50.5%)	151 (53.4%)		1422 (50.6%)	258 (51.9%)	
Heterozygous	1292 (42.7%)	113 (39.9%)		1203 (42.8%)	202 (40.6%)	
Homozygous	206 (6.8%)	19 (6.7%)		188 (6.7%)	37 (7.4%)	
GCKR (rs1260326 C > T) (*n*, %)			0.341 ^a^			0.101 ^a^
Wildtype	1017 (33.6%)	107 (37.8%)		939 (33.4%)	185 (37.2%)	
Heterozygous	1473 (48.7%)	131 (46%)		1385 (49.2%)	219 (44.1%)	
Homozygous	537 (17.7%)	45 (15.9%)		489 (17.4%)	93 (18.7%)	
LYPLAL1 (rs12137855 C > T) (*n*, %)			0.683 ^a^			0.767 ^a^
Wildtype	1880 (62.1%)	177 (62.5%)		1745 (62.0%)	312 (62.8%)	
Heterozygous	1006 (33.2%)	96 (33.9%)		942 (33.5%)	160 (32.2%)	
Homozygous	141 (4.7%)	10 (3.5%)		126 (4.5%)	25 (5.0%)	

^a^ Chi-square *p*-value; ^b^ Student’s *t*-test *p*-value.

**Table 4:** Association between MAFLD-related variants stratified by homo- and heterozygosity with MACE in Australian ASPREE participants with MAFLD.

	MACE Median Follow-Up 8.3 (7.3–9.4) Years					
MAFLD Variants	HR (95% CI)	*p*-Value	aHR * (95% CI)	*p*-Value	aHR ** (95% CI)	*p*-Value
PNPLA3 (rs738409 C > G)						
Wildtype	*Reference*	*–*	*Reference*	*–*	*Reference*	–
Heterozygous	0.87 (0.68–1.12)	0.275	0.87 (0.68–1.12)	0.281	0.88 (0.68–1.14)	0.332
Homozygous	0.88 (0.50–1.55)	0.654	0.92 (0.52–1.61)	0.761	0.91 (0.52–1.61)	0.747
MBOAT7 (rs641738 C > T)						
Wildtype	*Reference*	*–*	*Reference*	*–*	*Reference*	–
Heterozygous	0.82 (0.63–1.05)	0.119	0.81 (0.62–1.04)	0.103	0.81 (0.62–1.05)	0.111
Homozygous	0.68 (0.48–0.97)	**0.034**	0.67 (0.47–0.96)	**0.027**	0.64 (0.44–0.92)	**0.015**
TM6SF2 (rs58542926 C > T)						
Wildtype	*Reference*	*–*	*Reference*	*–*	*Reference*	–
Heterozygous	1.22 (0.89–1.66)	0.216	1.22 (0.89–1.66)	0.222	1.25 (0.91–1.72)	0.165
Homozygous	1.19 (0.29–4.82)	0.810	1.04 (0.26–4.25)	0.952	1.19 (0.29–4.85)	0.811
HSD17B13 (rs72613567 T > TA)						
Wildtype	*Reference*	–	*Reference*	*–*	*Reference*	–
Heterozygous	0.87 (0.69–1.13)	0.335	0.89 (0.70–1.14)	0.361	0.86 (0.67–1.10)	0.221
Homozygous	0.92 (0.57–1.48)	0.729	0.88 (0.54–1.42)	0.598	0.92 (0.57–1.49)	0.735
GCKR (rs1260326 C > T)						
Wildtype	*Reference*	–	*Reference*	*–*	*Reference*	–
Heterozygous	0.85 (0.66–1.10)	0.225	0.87 (0.67–1.12)	0.286	0.90 (0.69–1.16)	0.412
Homozygous	0.82 (0.58–1.17)	0.280	0.84 (0.59–1.19)	0.330	0.84 (0.58–1.20)	0.331
LYPLAL1 (rs12137855 C > T)						
Wildtype	*Reference*	–	*Reference*	*–*	*Reference*	–
Heterozygous	1.02 (0.80–1.31)	0.857	1.04 (0.81–1.33)	0.772	1.05 (0.82–1.36)	0.687
Homozygous	0.80 (0.42–1.52)	0.504	0.81 (0.42–1.53)	0.507	0.87 (0.46–1.66)	0.678

HR = hazard ratio; aHR = adjusted hazard ratio. * Adjusted for age and sex. ** ASPREE adjusted model [46] includes the following: age, sex, creatinine, HDL-C, non-HDL-C, systolic blood pressure, prescription of an antihypertensive medication(s), T2DM (self-described T2DM and/or a fasting blood glucose ≥ 126 mg/dL and/or hypoglycemic medication(s)), current smoking status, and trial treatment with aspirin or placebo. All models adjusted for 20 principal components.

**Table 5:** Association between MAFLD-related genetic variants and MACE in Australian ASPREE participants with MAFLD. Homozygotes and heterozygotes grouped.

	MACE Median Follow-Up 8.3 (7.3–9.4) Years					
MAFLD Genetic Variants	HR (95% CI)	*p*-Value	Model 1 aHR * (95% CI)	*p*-Value	Model 2 aHR ** (95% CI)	*p*-Value
PNPLA3 (rs738409 C > G)	0.87 (0.68–1.11)	0.251	0.88 (0.69–1.12)	0.283	0.88 (0.69–1.13)	0.327
MBOAT7 (rs641738 C > T)	0.78 (0.61–0.99)	**0.043**	0.77 (0.60–0.98)	**0.034**	0.76 (0.59–0.97)	**0.029**
TM6SF2 (rs58542926 C > T)	1.22 (0.90–1.65)	0.211	1.21 (0.89–1.64)	0.230	1.25 (0.91–1.70)	0.162
HSD17B13 (rs72613567 T > TA)	0.89 (0.70–1.13)	0.335	0.89 (0.70–1.13)	0.332	0.86 (0.68–1.10)	0.233
GCKR (rs1260326 C > T)	0.85 (0.66–1.08)	0.174	0.86 (0.68–1.10)	0.228	0.88 (0.69–1.13)	0.312
LYPLAL1 (rs12137855 C > T)	1.00 (0.78–1.27)	0.985	1.01 (0.79–1.29)	0.935	1.03 (0.81–1.32)	0.796

HR = hazard ratio; aHR = adjusted hazard ratio. * Model 1: Adjusted for age and sex. ** Model 2: ASPREE adjusted model [46] includes the following: age, sex, creatinine, HDL-C, non-HDL-C, systolic blood pressure, prescription of an antihypertensive medication(s), DM (self-described DM and/or a fasting blood glucose ≥7.0 mmol/L and/or hypoglycemic medication(s)), current smoking status, and trial treatment with aspirin or placebo. All models adjusted for 20 principal components.

**Table 6:** Association between MAFLD-related genetic variants stratified by homo- and heterozygosity with all-cause mortality in Australian ASPREE participants with MAFLD.

	All-Cause Mortality Median Follow-Up 8.4 (7.4–9.5) Years					
MAFLD Variants	HR (95% CI)	*p*-Value	aHR * (95% CI)	*p*-Value	aHR ** (95% CI)	*p*-Value
PNPLA3 (rs738409 C > G)						
Wildtype	*Reference*	*–*	*Reference*	*–*	*Reference*	–
Heterozygous	0.95 (0.79–1.14)	0.568	0.94 (0.78–1.14)	0.531	0.92 (0.76–1.12)	0.414
Homozygous	0.79 (0.49–1.22)	0.264	0.81 (0.52–1.28)	0.371	0.76 (0.47–1.21)	0.246
MBOAT7 (rs641738 C > T)						
Wildtype	*Reference*	*–*	*Reference*	*–*	*Reference*	–
Heterozygous	0.98 (0.80–1.21)	0.876	0.95 (0.77–1.16)	0.621	0.96 (0.78–1.18)	0.706
Homozygous	1.14 (0.89–1.47)	0.291	1.09 (0.85–1.41)	0.481	1.10 (0.85–1.42)	0.475
TM6SF2 (rs58542926 C > T)						
Wildtype	*Reference*	*–*	*Reference*	*–*	*Reference*	–
Heterozygous	1.26 (1.00–1.59)	0.053	1.22 (0.97–1.54)	0.093	1.19 (0.93–1.51)	0.166
Homozygous	0.97 (0.31–3.04)	0.958	0.84 (0.27–2.65)	0.769	0.91 (0.29–2.86)	0.868
HSD17B13 (rs72613567 T > TA)						
Wildtype	*Reference*	*–*	*Reference*	*–*	*Reference*	–
Heterozygous	0.92 (0.76–1.10)	0.368	0.91 (0.76–1.10)	0.346	0.89 (0.74–1.08)	0.243
Homozygous	1.05 (0.74–1.48)	0.798	0.98 (0.69–1.38)	0.887	0.94 (0.66–1.35)	0.750
GCKR (rs1260326 C > T)						
Wildtype	*Reference*	*–*	*Reference*	*–*	*Reference*	–
Heterozygous	0.81 (0.67–0.99)	0.037	0.83 (0.68–1.01)	0.210	0.86 (0.71–1.06)	0.156
Homozygous	0.96 (0.75–1.24)	0.758	0.97 (0.75–1.24)	0.786	0.96 (0.74–1.25)	0.761
LYPLAL1 (rs12137855 C > T)						
Wildtype	*Reference*	*–*	*Reference*	*–*	*Reference*	–
Heterozygous	0.97 (0.80–1.17)	0.736	1.00 (0.82–1.21)	0.989	0.93 (0.76–1.13)	0.439
Homozygous	1.12 (0.74–1.69)	0.591	1.11 (0.74–1.68)	0.603	1.09 (0.71–1.67)	0.695

HR = hazard ratio; aHR = adjusted hazard ratio. * Adjusted for age and sex. ** ASPREE adjusted model [47] includes the following: age, sex, baseline 3MS score, gait speed, grip strength, smoking status, eGFR, a CES-D-10 score of ≥8, baseline T2DM, and trial treatment with aspirin or placebo. All models adjusted for 20 principal components.

**Table 7:** Association between MAFLD-related genetic variants and mortality in Australian ASPREE participants with MAFLD. Homozygotes and heterozygotes grouped.

	All-Cause Mortality Median Follow-Up 8.4 (7.4–9.5) Years					
MAFLD Genetic Variants	HR (95% CI)	*p*-Value	Model 1 aHR * (95% CI)	*p*-Value	Model 2 aHR ** (95% CI)	*p*-Value
PNPLA3 (rs738409 C > G)	0.93 (0.77–1.11)	0.400	0.93 (0.77–1.11)	0.409	0.90 (0.75–1.09)	0.283
MBOAT7 (rs641738 C > T)	1.03 (0.85–1.24)	0.782	0.99 (0.82–1.20)	0.912	1.00 (0.82–1.21)	0.988
TM6SF2 (rs58542926 C > T)	1.25 (0.99–1.57)	0.061	1.20 (0.96–1.51)	0.115	1.17 (0.93–1.49)	0.188
HSD17B13 (rs72613567 T > TA)	0.94 (0.78–1.12)	0.465	0.92 (0.77–1.10)	0.379	0.90 (0.75–1.08)	0.256
GCKR (rs1260326 C > T)	0.85 (0.71–1.02)	0.081	0.86 (0.72–1.04)	0.113	0.89 (0.74–1.07)	0.222
LYPLAL1 (rs12137855 C > T)	0.99 (0.82–1.18)	0.876	1.01 (0.84–1.22)	0.890	0.94 (0.78–1.14)	0.549

HR = hazard ratio; aHR = adjusted hazard ratio. * Model 1: Adjusted for age and sex. ** Model 2: ASPREE adjusted model [47] includes the following baseline variables: age, sex, baseline 3MS score, gait speed, grip strength, smoking status, eGFR, a CES-D-10 score of ≥8, DM, and trial treatment with aspirin or placebo. All models adjusted for 20 principal components.

**Table 8:** Genetic variants and their associations with MACE and all-cause mortality in those without definite MAFLD (FLI < 60).

	MACE Median Follow-Up 8.4 (7.4–9.5) Years (Total MACE = 380; *n* = 5441)				All-Cause Mortality Median Follow-Up 8.4 (7.4–9.5) Years (Total Deaths = 775; *n* = 5441)			
MAFLD Genetic Variants	HR (95% CI)	*p*-Value	aHR * (95% CI)	*p*-Value	HR (95% CI)	*p*-Value	aHR ** (95% CI)	*p*-Value
PNPLA3 (rs738409 C > G)								
Wildtype	*Reference*	*–*	*Reference*	*–*	*Reference*	*–*	*Reference*	–
Heterozygous	1.06 (0.86–1.31)	0.590	1.12 (0.90–1.39)	0.303	1.03 (0.89–1.20)	0.686	1.05 (0.90–1.22)	0.564
Homozygous	1.29 (0.83–2.00)	0.257	1.41 (0.90–2.22)	0.134	0.92 (0.65–1.32)	0.665	1.07 (0.75–1.54)	0.696
MBOAT7 (rs641738 C > T)								
Wildtype	*Reference*	*–*	*Reference*	*–*	*Reference*	*–*	*Reference*	–
Heterozygous	1.03 (0.82–1.30)	0.768	1.06 (0.84–1.33)	0.632	*0.90 (0.77–1.05)*	0.180	0.90 (0.77–1.06)	0.221
Homozygous	0.96 (0.71–1.29)	0.772	0.92 (0.68–1.25)	0.598	*0.91 (0.74–1.12)*	0.382	0.85 (0.69–1.05)	0.127
TM6SF2 (rs58542926 C > T)								
Wildtype	*Reference*	*–*	*Reference*	*–*	*Reference*	*–*	*Reference*	–
Heterozygous	1.03 (0.78–1.38)	0.820	(0.75–1.35)	0.865	1.18 (0.97–1.43)	0.091	1.17 (0.96–1.43)	0.111
Homozygous	1.25 (0.31–5.05)	0.750	0.85 (0.12–6.12)	0.875	1.62 (0.67–3.93)	0.282	1.79 (0.66–4.82)	0.250
HSD17B13 (rs72613567 T > TA)								
Wildtype	*Reference*	*–*	*Reference*	*–*	*Reference*	*–*	*Reference*	–
Heterozygous	0.85 (0.69–1.06)	0.155	0.86 (0.69–1.08)	0.191	0.88 (0.76–1.03)	0.109	0.89 (0.76–1.04)	0.144
Homozygous	1.36 (0.97–1.90)	0.076	1.26 (0.89–1.80)	0.194	1.00 (0.77–1.30)	0.999	0.96 (0.72–1.26)	0.747
GCKR (rs1260326 C > T)								
Wildtype	*Reference*	*–*	*Reference*	*–*	*Reference*	*–*	*Reference*	–
Heterozygous	1.18 (0.87–1.60)	0.287	1.13 (0.83–1.55)	0.447	*0.96 (0.83–1.12)*	0.629	0.99 (0.84–1.16)	0.866
Homozygous	0.99 (0.72–1.37)	0.962	0.99 (0.71–1.37)	0.939	*0.96 (0.77–1.19)*	0.709	1.06 (0.85–1.33)	0.595
LYPLAL1 (rs12137855 C > T)								
Wildtype	*Reference*	*–*	*Reference*	*–*	*Reference*	*–*	*Reference*	–
Heterozygous	1.01 (0.63–1.62)	0.964	1.06 (0.65–1.74)	0.817	1.05 (0.91–1.23)	0.490	1.06 (0.91–1.23)	0.484
Homozygous	0.87 (0.55–1.37)	0.545	0.91 (0.56–1.48)	0.704	0.91 (0.64–1.29)	0.581	0.98 (0.68–1.40)	0.902

* ASPREE adjusted model [46] includes the following: age, sex, creatinine, HDL-C, non-HDL-C, systolic blood pressure, prescription of an antihypertensive medication(s), DM (self-described DM and/or a fasting blood glucose ≥ 7.0 mmol/L and/or hypoglycemic medication(s)), current smoking status, and trial treatment with aspirin or placebo. ** ASPREE adjusted model [47] includes the following: age, sex, baseline 3MS score, gait speed, grip strength, smoking status, eGFR, a CES-D-10 score of ≥8, baseline DM, and trial treatment with aspirin or placebo. All models adjusted for 20 principal components.

**Table 9:** Genetic variants and their associations with MACE and all-cause mortality in those with MASLD.

	MACE Median Follow-Up 8.3 (7.3–9.4) Years (Total MACE = 216; *n* = 2624)				All-Cause Mortality Median Follow-Up 8.4 (7.4–9.5) Years (Total Deaths = 374, *n* = 2624)			
MASLD Genetic Variants	HR (95% CI)	*p*-Value	aHR * (95% CI)	*p*-Value	HR (95% CI)	*p*-Value	aHR ** (95% CI)	*p*-Value
PNPLA3 (rs738409 C > G)								
Wildtype	*Reference*	*–*	*Reference*	*–*	*Reference*	*–*	*Reference*	–
Heterozygous	0.83 (0.62–1.11)	0.207	0.84 (0.62–1.13)	0.250	0.92 (0.74–1.14)	0.424	0.91 (0.72–1.13)	0.380
Homozygous	1.12 (0.64–1.94)	0.686	1.17 (0.67–2.04)	0.582	0.81 (0.50–1.31)	0.384	0.77 (0.47–1.27)	0.310
MBOAT7 (rs641738 C > T)								
Wildtype	*Reference*	*–*	*Reference*	*–*	*Reference*	*–*	*Reference*	–
Heterozygous	0.82 (0.61–1.10)	0.185	0.79 (0.58–1.06)	0.115	*1.00 (0.79–1.26)*	0.976	0.94 (0.73–1.19)	0.599
Homozygous	0.66 (0.44–0.99)	**0.044**	0.59 (0.38–0.90)	**0.013**	*1.08 (0.81–1.45)*	0.596	1.03 (0.77–1.39)	0.838
TM6SF2 (rs58542926 C > T)								
Wildtype	*Reference*	*–*	*Reference*	*–*	*Reference*	*–*	*Reference*	–
Heterozygous	1.19 (0.83–1.79)	0.354	1.21 (0.84–1.75)	0.310	1.42 (1.09–1.84)	**0.008**	1.31 (0.99–1.71)	0.056
Homozygous	0.87 (0.12–6.27)	0.890	0.97 (0.13–7.09)	0.978	1.02 (0.25–4.14)	0.976	0.91 (0.22–3.71)	0.893
HSD17B13 (rs72613567 T > TA)								
Wildtype	*Reference*	*–*	*Reference*	*–*	*Reference*	*–*	*Reference*	–
Heterozygous	0.84 (0.63–1.11)	0.220	0.81 (0.60–1.08)	0.151	0.92 (0.74–1.13)	0.420	0.90 (0.72–1.12)	0.351
Homozygous	0.96 (0.57–1.63)	0.886	0.97 (0.57–1.65)	0.919	1.00 (0.67–1.50)	0.999	0.91 (0.60–1.39)	0.665
GCKR (rs1260326 C > T)								
Wildtype	*Reference*	*–*	*Reference*	*–*	*Reference*	*–*	*Reference*	–
Heterozygous	0.79 (0.59–1.05)	0.108	0.84 (0.62–1.13)	0.240	*0.89 (0.71–1.12)*	0.339	0.95 (0.75–1.20)	0.672
Homozygous	0.70 (0.47–1.06)	0.091	0.73 (0.48–1.11)	0.136	*1.00 (0.75–1.34)*	0.987	1.02 (0.75–1.39)	0.878
LYPLAL1 (rs12137855 C > T)								
Wildtype	*Reference*	*–*	*Reference*	*–*	*Reference*	*–*	*Reference*	–
Heterozygous	1.13 (0.85–1.51)	0.389	1.19 (0.89–1.59)	0.243	1.06 (0.85–1.33)	0.577	1.02 (0.82–1.29)	0.835
Homozygous	1.10 (0.58–2.10)	0.776	1.18 (0.61–2.25)	0.625	1.37 (0.88–2.13)	0.161	1.27 (0.80–2.02)	0.307

* ASPREE adjusted model [46] includes the following: age, sex, creatinine, HDL-C, non-HDL-C, systolic blood pressure, prescription of an antihypertensive medication(s), DM (self-described DM and/or a fasting blood glucose ≥ 7.0 mmol/L and/or hypoglycemic medication(s)), current smoking status, and trial treatment with aspirin or placebo. ** ASPREE adjusted model [47] includes the following: age, sex, baseline 3MS score, gait speed, grip strength, smoking status, eGFR, a CES-D-10 score of ≥8, baseline DM, and trial treatment with aspirin or placebo. All models adjusted for 20 principal components.

## Data Availability

The datasets used and/or analyzed for this publication are available via the ASPREE Principal Investigators. Requests for data access can be directed to aspree.ams@monash.edu.
